# Global research trends and emerging frontiers in young-onset stroke: a bibliometric analysis (1996–2026)

**DOI:** 10.3389/fneur.2026.1849454

**Published:** 2026-08-03

**Authors:** Bowen Zheng, Zifeng Dai, Li Sun, Yujia Sun, Shan Cong, Tao Yu, Meng Wang, Yulin Qian

**Affiliations:** 1 First Teaching Hospital of Tianjin University of Traditional Chinese Medicine, Tianjin, China; 2 National Clinical Research Center for Chinese Medicine, Tianjin, China; 3 Graduate Department, Tianjin University of Traditional Chinese Medicine, Tianjin, China; 4 Tianjin Xiqing District Traditional Chinese Medicine Hospital, Tianjin, China

**Keywords:** bibliometrics, CiteSpace, stroke, VOSviewer, young adults, young-onset

## Abstract

**Objective:**

Young-onset stroke (YOS) has increasing public health relevance. This study mapped global research trends, hotspots, and emerging frontiers in YOS from 1996 to 2026.

**Methods:**

English articles and reviews from 1996 to 2026 were retrieved from the Web of Science Core Collection, yielding 5,249 records after screening. PubMed was analyzed for external validation, with 2,929 eligible records. bibliometrix, VOSviewer, and CiteSpace were used to analyze publication output, collaboration networks, keywords, bursts, and co-citations. A strict title-field subset evaluated search-strategy sensitivity.

**Results:**

Publication output increased markedly after 2018. The United States and China were the leading contributors. Major topics included ischemic stroke, risk factors, cardiovascular comorbidity, etiologic evaluation, acute treatment, rehabilitation, and survivorship. Recent bursts indicated growing attention to disease burden, mechanical thrombectomy, medical therapy, stroke survivors, quality of life, fatigue, unmet needs, and machine learning. PubMed validation and strict title-field sensitivity analysis supported the main temporal and thematic patterns.

**Conclusion:**

YOS research has expanded into a multidimensional field. Future work should prioritize harmonized definitions, multinational data resources, age-specific etiologic and treatment studies, long-term cognitive and psychosocial outcomes, and rehabilitation.

## Introduction

Stroke is a leading cause of death and long-term disability worldwide. It imposes substantial societal and economic burdens (1). While stroke has historically been seen in older adults, new epidemiological studies suggest increasing or non-declining stroke burden among younger adults in several populations (2, 3). In the literature, young-onset stroke is often defined as occurring between ages 15–49 or 18–50, depending on data source and study purpose (2, 4). Interest in young-onset stroke is growing, partly due to its life-course impacts. Compared with late-life stroke, younger patients live longer with disability, face disruptions in education and work, and place sustained demands on caregivers and health systems (2).

Notably, recent epidemiological reviews show a changing landscape over two decades. Trends in younger age groups differ from those in older populations, and there are major disparities between high-income and low- and middle-income areas (2, 5). These findings underscore the need for focused research on young-onset stroke for several reasons. First, the preventable burden may be substantial because risk exposure and prevention extend across decades. Second, etiologic evaluation and management often differ from those in older patients. Third, long-term outcomes, including recurrent vascular events, psychosocial effects, and cognitive changes, are particularly important in patients with longer life expectancy (2, 6).

Young-onset stroke shows substantial clinical heterogeneity. Younger patients may present with ischemic or hemorrhagic stroke, and clinical severity, recovery, and long-term neuropsychological outcomes can vary widely. The etiologic spectrum is also broader than in older groups. Alongside vascular risk factors (e.g., hypertension, diabetes, dyslipidemia, smoking), younger patients more often have causes like cervical artery dissection, cardioembolism, thrombophilia, autoimmune vasculopathies, and cryptogenic mechanisms. Management of contributors like patent foramen ovale in cryptogenic stroke has changed over time, highlighting a need to improve patient selection and evidence translation for younger cohorts (6).

Large-scale assessments—for example, those using Global Burden of Disease evaluations and major policy commissions—quantify stroke burden and its unequal distribution by region and socioeconomic status (1, 7). However, these broad analyses prioritize population-level comparisons. They may not capture how research focus, collaboration networks, or thematic emphases have changed in young-onset stroke over time (1, 2).

Despite growth in publications, long-term global mapping—covering knowledge base, collaboration, and emerging themes—is limited (2). Bibliometric mapping focused on young-onset stroke is still rare, especially compared to wider stroke research (8). This leaves uncertainty about how research priorities and key knowledge clusters have evolved in this age group. Bibliometric and visual methods help fill this gap. They summarize publication growth, contributors, collaborative networks, co-citation knowledge bases, and burst signals that point to fast-emerging topics.

Therefore, we conducted a bibliometric and visual analysis of young-onset stroke research in the Web of Science Core Collection (WoSCC) from 1996 to 2026. We performed a descriptive analysis of publication and citation trends. We used science-mapping techniques: collaboration and co-occurrence network visualization (VOSviewer), co-citation and burst detection (CiteSpace), and bibliometric statistics (bibliometrix). Our study aims to provide a detailed overview of the field. We identify leading contributors, sources, and collaborations, outline the core knowledge base, and highlight emerging frontiers to inform research planning and future evidence synthesis in young-onset stroke (9, 10). To address database-specific bias and improve robustness, PubMed was used as an external validation dataset to examine the cross-database consistency of annual publication trends and keyword-level thematic patterns identified in WoSCC. A search-strategy sensitivity analysis based on a stricter title-field subset was further performed to evaluate the stability of the main bibliometric findings.

## Materials and methods

### Study design

This study used a bibliometric and visualization design to examine global young-onset stroke (YOS) research from 1996 to 2026. It mapped publication output, collaboration patterns, source networks, keyword structures, co-cited references, and temporal research fronts. WoSCC served as the primary science-mapping dataset, and PubMed served as an independent biomedical validation dataset. The two datasets were processed separately and were not merged.

### Data sources

WoSCC was selected as the primary source because it provides standardized metadata on cited references, citations, journals, authors, affiliations, and document types. These fields are required for co-citation, collaboration, and source-network analyses. PubMed was used for external validation because of its biomedical coverage and controlled vocabulary indexing. However, PubMed does not provide the same bibliometric metadata structure as WoSCC and is not directly comparable for combined co-citation or total-link-strength analyses. PubMed records were therefore used to assess whether the keyword and temporal structures found in WoSCC could be reproduced in an independent biomedical database.

Scopus was not merged into the main analysis because the study aimed to construct a reproducible dataset with internally consistent cited-reference, citation, journal, author, affiliation, and document-type fields. Scopus has broad journal coverage. However, combining Scopus and WoSCC may introduce heterogeneity in reference formats, document-type labels, institutional names, citation counts, and duplicate-record identification. Such heterogeneity can affect co-citation networks, total link strength, centrality estimates, and cluster detection. Google Scholar was not used because its limitations in bulk export, deduplication, and reproducibility make it unsuitable for a transparent bibliometric workflow (11, 12).

### Search strategy

Both databases were searched on June 12, 2026, and the time window was 1996 through 2026. The WoSCC searches were conducted with “Web of Science Core Collection” selected under “Search in” and “All” selected under “Editions.” Only English-language records were included. Complete search strings are provided in the Supplementary material. The WoSCC strategy used the Topic field. Stroke-related terms included stroke, strokes, cerebrovascular accident or event, cerebral or brain infarction, cerebral or brain ischemia/ischaemia, ischemic/ischaemic stroke, intracerebral or cerebral hemorrhage/haemorrhage, hemorrhagic/haemorrhagic stroke, subarachnoid hemorrhage/haemorrhage, and cerebral venous or cerebral venous sinus thrombosis. Age-related terms included young, younger, young-onset, early-onset, young adult, young people, young patient, younger population, aged 15–49, aged 18–49, aged 18–50, under 50, and younger than 50.

For WoSCC, stroke/cerebrovascular concepts and young-age concepts were linked using the NEAR/10 operator or explicit young-stroke phrases. For PubMed, a sensitive Boolean combination of MeSH and Title/Abstract terms was supplemented by database-specific proximity expressions, followed by publication-type filtering and manual topical eligibility screening.

### Eligibility criteria

Eligible records had to focus substantively on stroke or cerebrovascular disease in young adults or younger populations. Eligible topics included etiology, risk factors, mechanisms, diagnosis, acute treatment, secondary prevention, prognosis, recurrence, mortality, rehabilitation, quality of life, cognitive outcomes, psychiatric outcomes, survivorship, and disease burden in YOS. Studies including mixed-age stroke populations were eligible only when young adults or young-onset stroke patients were analyzed separately, highlighted as a subgroup, or central to the research question. Records were excluded during post-retrieval screening if they focused on non-stroke young-onset diseases, non-neurological meanings of stroke such as heat stroke or stroke volume, or records in which stroke appeared only as a peripheral background endpoint. Records with insufficient information in the title or abstract were manually reviewed using available metadata before the final eligibility decision.

### Study selection and data management

The WoSCC topic-field search identified 8,966 records. After filters for year, language, and document type, 6,063 English-language articles or reviews published from 1996 through 2026 were retrieved. After NoteExpress-assisted deduplication, two reviewers independently screened titles and abstracts against the predefined eligibility criteria. Disagreements were resolved through discussion or adjudication by a senior reviewer. After screening, 5,249 WoSCC records were retained. All eligible WoSCC records were exported in plain-text format with full records and cited references.

PubMed does not provide a single publication type equivalent to the WoSCC Article category. Original research articles were identified using a combined publication-type and topic-based approach. Records indexed as Journal Article, Clinical Trial, Observational Study, Comparative Study, Multicenter Study, Randomized Controlled Trial, Validation Study, or related research categories were retained when topic criteria were met. Reviews, systematic reviews, and meta-analyses were also retained because the study scope included articles and reviews. Editorials, letters, comments, news items, corrections, retractions, books, and book chapters were excluded. The same post-retrieval topical screening criteria were then applied to PubMed records.

**Figure 1:**
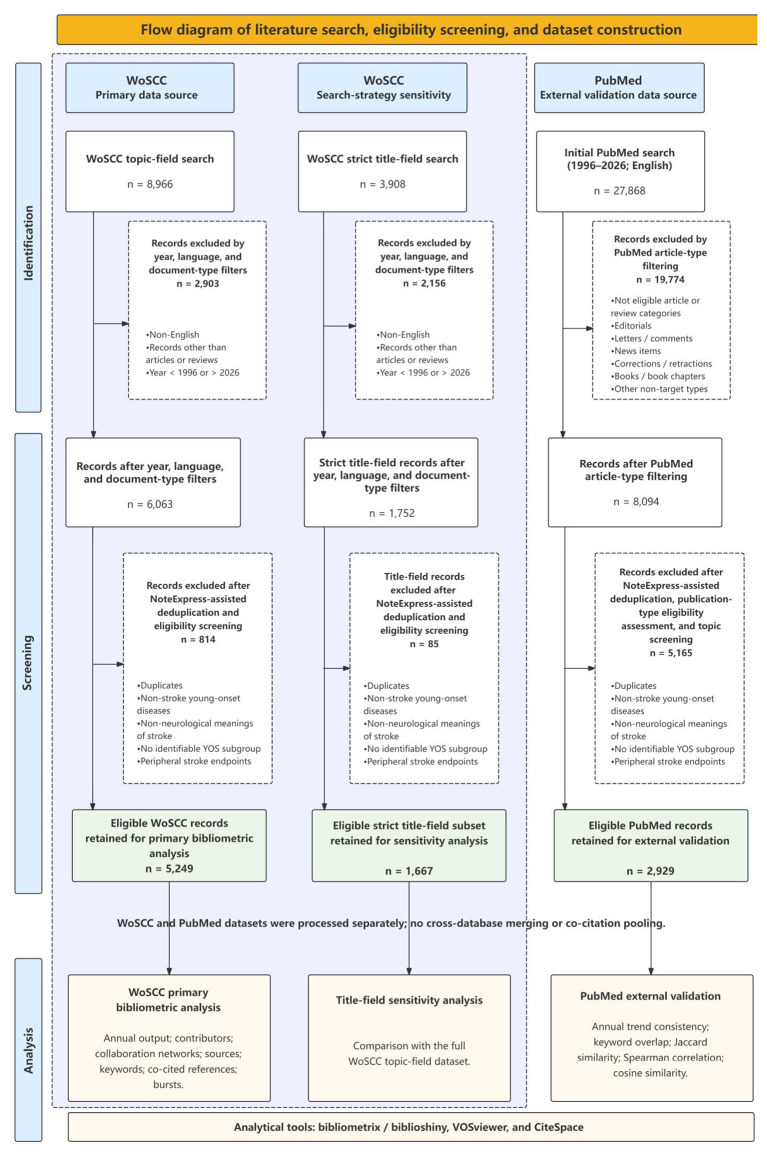
Flow diagram of literature search, eligibility screening, and dataset construction for the WoSCC main analysis, strict title-field sensitivity analysis, and PubMed external validation.

The initial PubMed search retrieved 27,868 records. Following article-type filtering, 8,094 records remained for validation screening. After NoteExpress-assisted deduplication and manual screening for publication-type and topic eligibility, 2,929 records were retained. PubMed records were exported separately in a format compatible with keyword extraction and validation analyses. The selection process is summarized in a PRISMA-style flow diagram (Figure 1).

### Data cleaning and normalization

Names and terms were standardized before analysis to reduce artificial fragmentation. Keyword variants were harmonized, including ischemic/ischaemic stroke, hemorrhage/haemorrhage, risk factor/risk factors, young adult/young adults, and young-onset/young onset. Institutional names were standardized by merging clear synonyms and subunits when they referred to the same organization.

Author disambiguation used surname and initials, full author names when available, institutional affiliation, ORCID information, and co-author context. Ambiguous cases were not forcibly merged. Geographic fields were analyzed according to the country/region information present in the database records. For example, records indexed as Mainland China, Hong Kong, Macau, or Taiwan were harmonized into the China category for aggregate bibliometric reporting. The same keyword-normalization principles were applied to PubMed validation records where applicable.

### Bibliometric indicators and visualization analysis

Bibliometric visualization was performed using the bibliometrix package in R version 4.4.3 (R Foundation for Statistical Computing, Vienna, Austria), VOSviewer version 1.6.20 (Centre for Science and Technology Studies (CWTS), Leiden University, Leiden, The Netherlands), and CiteSpace version 6.4.R2 (Chaomei Chen, Drexel University, Philadelphia, PA, United States). Annual publication counts were used to describe growth. Productivity referred to the number of publications. Total citations referred to WoSCC citation counts, whereas local citations referred to citations received within the retrieved dataset. Co-citation referred to the frequency with which two references were cited together.

The bibliometrix package and biblioshiny were used for descriptive statistics and thematic exploration of the primary WoSCC dataset. VOSviewer was used to visualize collaboration networks, source-level networks, and keyword co-occurrence. Total link strength (TLS), as exported by VOSviewer, quantified the total strength of co-authorship, co-occurrence, or citation links for a node. TLS was not interpreted as CiteSpace betweenness centrality.

CiteSpace was used for co-citation analysis, keyword clustering, burst detection, and knowledge mapping. The main settings were as follows: time slicing = 1996–2026; years per slice = 1; pruning = Pathfinder; node types = author, institution, country/region, keyword, and reference; and selection criterion = g-index (k = 25). Modularity *Q* and silhouette *S* were used to evaluate cluster structure and homogeneity (9).

### Validation and sensitivity analyses

For external validation, the PubMed dataset was processed separately using VOSviewer for keyword co-occurrence mapping and CiteSpace for keyword clustering. The same timespan and comparable parameter settings were used. Cross-database consistency was assessed by comparing high-frequency keywords, central nodes, and major cluster labels between WoSCC and PubMed. These comparisons were quantified using annual trend correlations, top-30 and top-50 keyword overlap rates, Jaccard similarity, Spearman rank correlation among shared high-frequency keywords, and cosine similarity.

Search-strategy sensitivity was evaluated by comparing the full WoSCC topic-field dataset with a strict title-field subset constructed using the same conceptual search logic. The full topic-field search initially identified 8,966 records, of which 5,249 were eligible for the main analysis after database filtering and screening. The strict title-field search initially identified 3,908 records; after applying the same year, language, and document-type filters, 1,752 records remained for screening, and 1,667 eligible records were retained.

### Ethics

This study used publicly available bibliographic data and did not involve individual patient data, human participants, or animal experiments. Ethical approval was not required.

## Results

### Overall characteristics of the dataset

A total of 5,249 articles and reviews were included in the analysis. The records covered young-onset stroke research from 1996 through 2026 and were produced by 25,510 authors from 2,971 institutions in 132 countries or regions. They were published in 1,128 sources. The included records received an average of 33.49 WoSCC citations per document. Annual output showed an overall upward trajectory, reached its highest annual level in 2022, and remained high through 2025. The 2026 count should be interpreted as a partial-year value.

### Basic characteristics

#### Annual publication output

**Figure 2:**
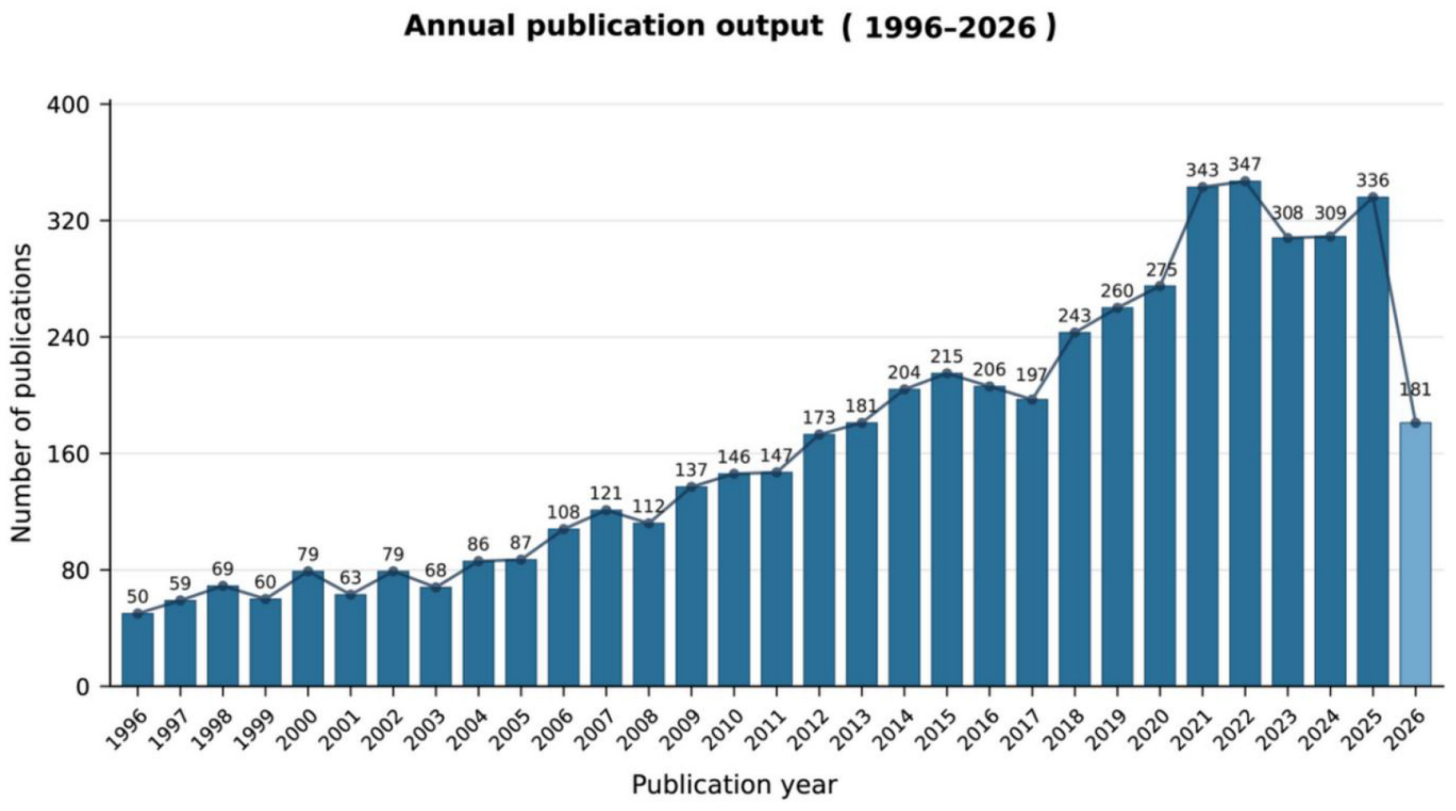
Annual publication output of young-onset stroke research from 1996 through 2026. The 2026 count represents an incomplete publication year.

Figure 2 shows the annual publication output from 1996 through 2026. From 1996 to 2006, output rose from 50 to 108 records, with an average of 73.5 publications per year. From 2007 to 2018, output increased from 121 to 243 records, with fluctuations, and the average during this period was 173.5 publications per year. Annual output first exceeded 200 records in 2014. From 2019 to 2025, the field entered a higher-output phase. The mean output during 2019–2025 was 4.2 times that during 1996–2006. Annual output remained above 300 records from 2021 through 2025, peaked at 347 publications in 2022, and remained high at 336 publications in 2025. The year 2026 contained 181 publications and represented an incomplete publication year.

### Country/region contributions, research areas, and international collaboration

**Figure 3:**
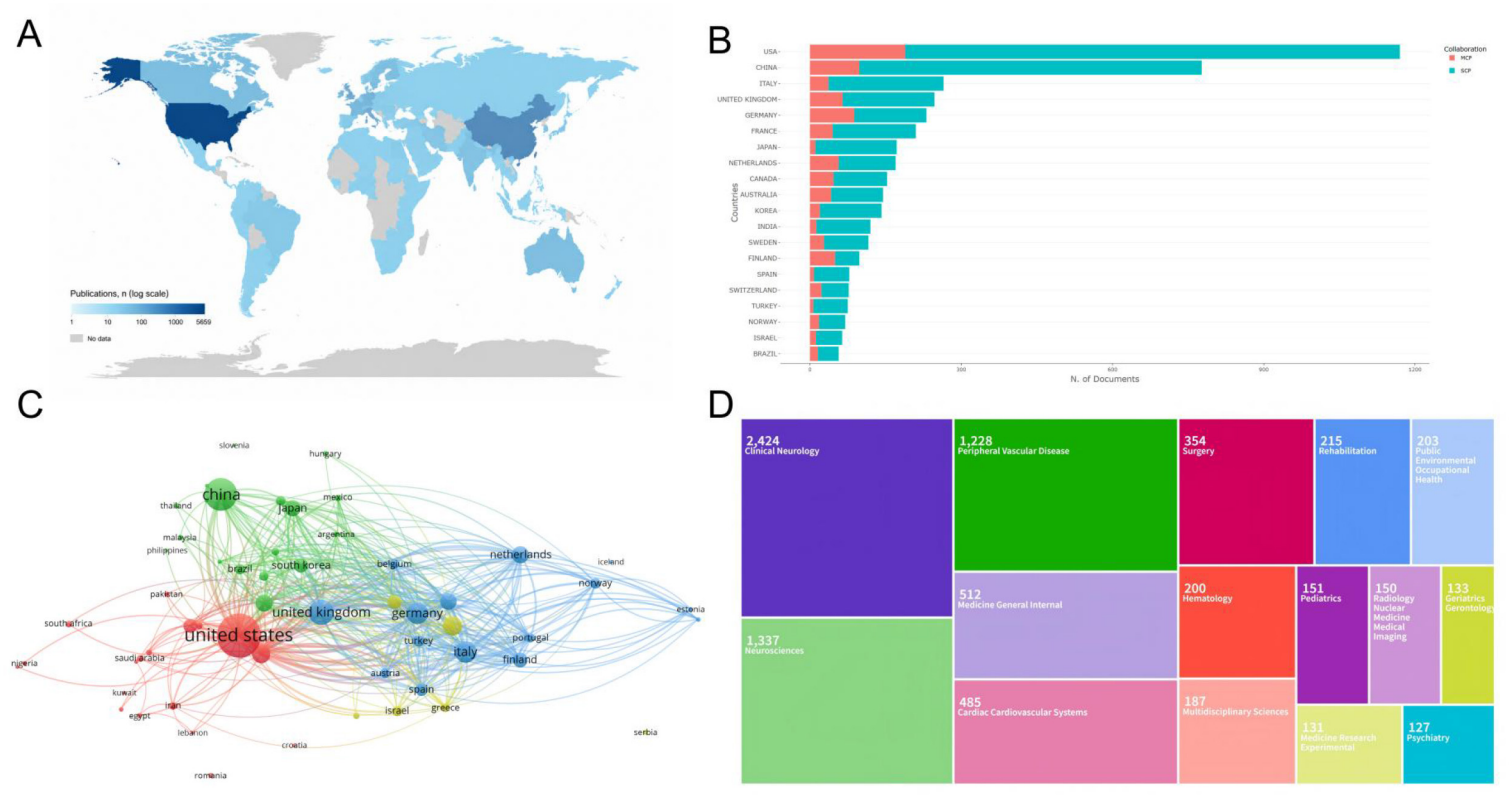
Geographic distribution, international collaboration, and research areas. **(A)** Global distribution of publications by country/region. Gray indicates no data, and publication counts are shown on a logarithmic scale. **(B)** Composition of publications by corresponding-author collaboration type in the leading countries, including single-country publications (SCP) and multiple-country publications (MCP). **(C)** Country/region collaboration network; node size reflects publication output, and line thickness indicates collaboration strength. **(D)** Treemap of major WoSCC research-area categories; rectangle size reflects the number of records in each category.

A total of 132 countries or regions contributed to young-onset stroke research. For aggregate reporting, records indexed as Mainland China, Hong Kong, Macau, or Taiwan were harmonized into the China category. The United States and China were the two largest contributors. Other high-output areas were distributed across Europe, North America, and the Asia-Pacific region. Figure 3A shows the geographic distribution of publications on a logarithmic scale, and Figure 3B shows single-country and multiple-country publications. The United States and China ranked highest for both indicators, showing that their leading output reflected both domestic productivity and international collaboration. Figure 3C presents the country/region collaboration network, which shows visible connections among North American, European, and Asia-Pacific contributors. Figure 3D summarizes the distribution of WoSCC research-area categories, with clinical neurology, neurosciences, and peripheral vascular disease as prominent categories.

### Institutional collaboration

A total of 2,971 standardized institutions contributed to the dataset. For the VOSviewer institutional collaboration analysis, the minimum threshold was 25 documents per institution. Seventy-nine institutions met this threshold. After disconnected items were excluded, the displayed network contained 78 items, five clusters, and 812 links. The five most productive institutions were Harvard University (180 records), the University of London (161), the University of California System (161), the University of Helsinki (154), and Helsinki University Hospital or HUS (143).

**Figure 4:**
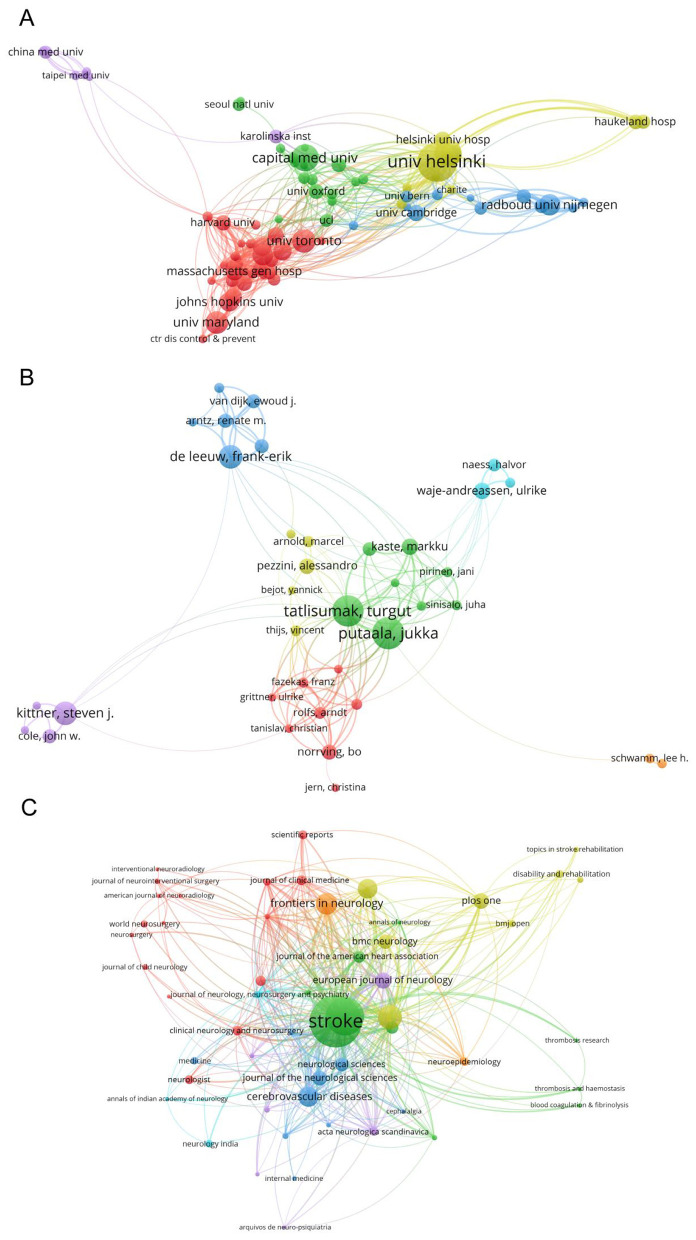
Collaboration and source networks in the WoSCC dataset. **(A)** Institutional collaboration network. **(B)** Author co-authorship network. **(C)** Source citation network. Node size reflects the item weight in the corresponding VOSviewer analysis, colors indicate network clusters, and links indicate collaboration, co-authorship, or citation relationships, respectively. Thicker links indicate stronger pairwise link strength.

In the VOSviewer collaboration network, the highest total link strength values were observed for the University of Helsinki (TLS = 259), Sahlgrenska University Hospital (199), the University of Gothenburg (198), Helsinki University Hospital (158), and Massachusetts General Hospital (125). These institutions occupied highly connected positions in the network. The institutional collaboration map in Figure 4A showed a multicenter structure rather than isolated single-center activity. Visible clusters involved Nordic stroke groups, Dutch and Western European institutions, North American universities, and several Chinese or Asia-Pacific institutions. These patterns suggest that institutional collaboration was organized around several regional and transnational research communities.

### Journals (sources): citation-based and productivity-based rankings

A total of 1,128 sources contributed to publications. The journal landscape showed a broad but relatively concentrated distribution. Specialized stroke and neurology journals dominated publication output and source-level network connectivity, whereas general medical and cardiovascular journals occupied prominent positions in citation-based rankings. For the VOSviewer source analysis, citation analysis was performed with sources as the unit of analysis and full counting as the counting method. The minimum threshold was set at 15 documents per source. Fifty-five sources met the threshold. After disconnected sources were excluded, the displayed network contained 54 items, seven clusters, and 835 links.

In terms of productivity (Table 1), Stroke ranked first with 362 articles, followed by Journal of Stroke and Cerebrovascular Diseases (248), Neurology (138), Frontiers in Neurology (121), and Cerebrovascular Diseases (110). In the citation-based ranking, Stroke also ranked first with 23,475 citations, followed by Neurology (7,556), The New England Journal of Medicine (4,724), The Lancet (3,911), and Circulation (3,884). The source network in Figure 4C showed that Stroke had the highest total link strength (TLS = 2,772). Other highly connected sources included Neurology (1,295), Journal of Stroke and Cerebrovascular Diseases (949), European Journal of Neurology (741), and Cerebrovascular Diseases (545). Most leading sources in the citation-based ranking were classified as Q1 in the 2025 Journal Citation Reports. These findings show that young-onset stroke research was disseminated through both core specialty journals and high-impact general medical, cardiovascular, and neurological journals.

**Table 1:** Top 10 journals by citation impact and publication output.

A. Citation-based ranking				
Rank	Journal	Citations	JIF	JCR quartile
1	Stroke	23,475	8.9	Q1
2	Neurology	7,556	8.5	Q1
3	N Engl J Med	4,724	78.5	Q1
4	Lancet	3,911	88.5	Q1
5	Circulation	3,884	38.6	Q1
6	Lancet Neurol	2,617	45.5	Q1
7	Cerebrovasc Dis	2,551	1.5	Q3
8	J Neurol Neurosurg Psychiatry	2,287	7.5	Q1
9	JAMA	2,244	55	Q1
10	J Am Coll Cardiol	1,884	22.3	Q1
B. Publication-based ranking				
Rank	Journal	Articles	JIF	JCR quartile
1	Stroke	362	8.9	Q1
2	J Stroke Cerebrovasc Dis	248	2.1	Q3
3	Neurology	138	8.5	Q1
4	Front Neurol	121	2.8	Q2
5	Cerebrovasc Dis	110	1.5	Q3
6	Int J Stroke	107	8.7	Q1
7	Eur J Neurol	83	3.9	Q1
8	PLoS One	81	2.6	Q2
9	J Neurol Sci	76	3.2	Q2
10	BMC Neurol	73	2.2	Q3

Citation counts refer to WoSCC citations to publications in the retrieved dataset. Journal Impact Factor (JIF) and JCR quartile are from JCR 2025.

### Core authors (influence, productivity, h-index, and collaboration)

A total of 25,510 authors contributed to young-onset stroke research. The leading authors were mainly from Finland, the Netherlands, China, the United States, Italy, and Norway, indicating a broad authorship base with a concentrated core group of influential contributors. For the author collaboration analysis, VOSviewer used co-authorship analysis with authors as the unit of analysis. Full counting was applied. Documents with more than 25 authors were excluded from the network calculation. A minimum threshold of 15 documents per author was used. Thirty-seven author items met this threshold. After disconnected items were excluded, the displayed network contained 36 items, seven clusters, and 142 links.

In terms of local citation impact (Table 2), Turgut Tatlisumak ranked first with 1,452 local citations, followed by Frank-Erik de Leeuw (1,352) and Jukka Putaala (1,307). In terms of publication output, Jukka Putaala ranked first with 105 articles, followed by Turgut Tatlisumak (99), Frank-Erik de Leeuw (75), Yongjun Wang (75), and Alessandro Pezzini (70). The field-specific author h-index was highest for Turgut Tatlisumak (35), followed by Jukka Putaala and Steven J. Kittner (34 each) and Frank-Erik de Leeuw (31).

**Table 2:** Top 10 authors by local citation impact and publication output.

A. Ranking by local citation impact				
Rank	Author	Local citations	Country	Institution
1	Turgut Tatlisumak	1,452	Finland	Helsinki University Hospital/HUS
2	Frank-Erik de Leeuw	1,352	Netherlands	Radboud University Medical Center
3	Jukka Putaala	1,307	Finland	Helsinki University Hospital/HUS
4	Ewoud J. van Dijk	835	Netherlands	Radboud University Medical Center
5	Elena Haapaniemi	794	Finland	Helsinki University Hospital/HUS
6	Markku Kaste	709	Finland	Helsinki University Hospital/HUS
7	Loes C.A. Rutten-Jacobs	657	Netherlands	Radboud University Medical Center
8	Steven J. Kittner	642	United States	University of Maryland Baltimore
9	Renate M. Arntz	606	Netherlands	Radboud University Medical Center
10	Didier Leys	604	France	Lille University Hospital
B. Ranking by publication output				
Rank	Author	Articles	Country	Institution
1	Jukka Putaala	105	Finland	Helsinki University Hospital/HUS
2	Turgut Tatlisumak	99	Finland	Helsinki University Hospital/HUS
3	Frank-Erik de Leeuw	75	Netherlands	Radboud University Medical Center
4	Yongjun Wang	75	China	Beijing Tiantan Hospital
5	Alessandro Pezzini	70	Italy	University of Brescia
6	Steven J. Kittner	66	United States	University of Maryland Baltimore
7	Ulrike Waje-Andreassen	50	Norway	Haukeland University Hospital
8	Halvor Naess	44	Norway	Haukeland University Hospital
9	Yu Zhang	41	China	Ruijin Hospital
10	Renate M. Arntz	40	Netherlands	Radboud University Medical Center

Local citations refer to citations received within the retrieved WoSCC dataset. Country and institution fields are based on standardized author-affiliation records.

The author collaboration network in Figure 4B showed several visible collaboration clusters rather than a fully integrated structure. Turgut Tatlisumak had the highest total link strength (TLS = 292), followed by Jukka Putaala (287), Frank-Erik de Leeuw (133), Arndt Rolfs (124), and Franz Fazekas and Markku Kaste (116 each). Prominent clusters involved Nordic collaborators, Dutch stroke-related groups, and North American collaborators. Additional links with Italian, Norwegian, Chinese, and other European contributors indicated cross-national collaboration. The overall structure was still dominated by a limited number of core teams.

### Knowledge mapping: hotspots, thematic structure, and emerging trends

**Figure 5:**
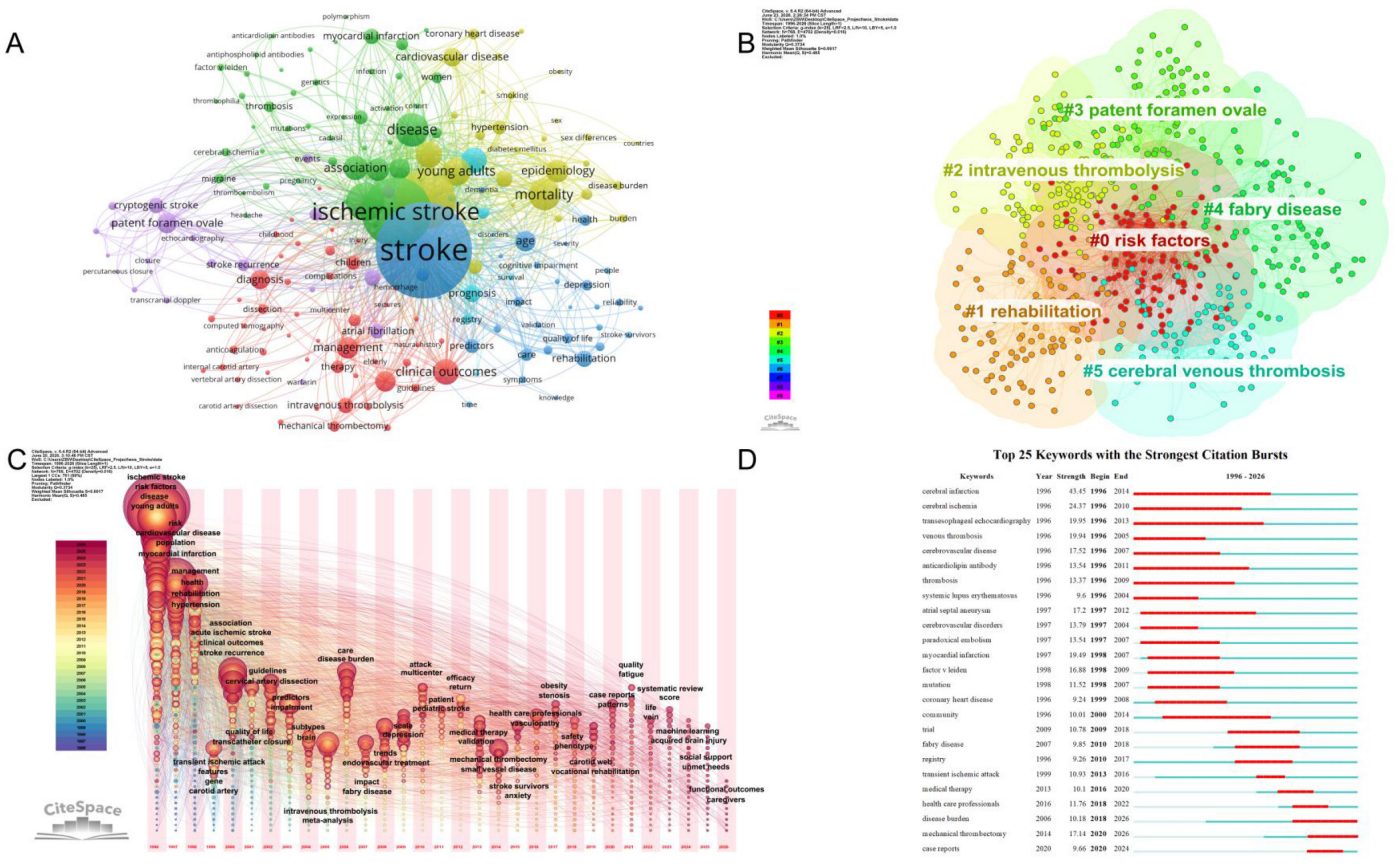
Keyword co-occurrence, clustering, temporal evolution, and burst analysis in the WoSCC dataset. **(A)** VOSviewer keyword co-occurrence network; node size reflects keyword occurrence, colors indicate co-occurrence clusters, and links indicate keyword co-occurrence relationships. **(B)** CiteSpace keyword clustering map; nodes are displayed with uniform size, and links indicate co-occurrence relationships. **(C)** CiteSpace keyword time-zone map showing the temporal evolution of keywords; node color corresponds to the time-slice scale shown in the legend, node size reflects keyword occurrence frequency, and links indicate keyword co-occurrence relationships across time slices. **(D)** Top 25 keywords with the strongest citation bursts; red bars indicate burst periods.

Keyword analysis outlined hotspots and thematic evolution in young-onset stroke research. For keyword co-occurrence mapping, full counting was applied, and the minimum occurrence threshold was set at 40. The resulting map contained 188 items, six clusters, and 10,320 links (Figure 5A). Overall, the field was organized around ischemic stroke and cerebrovascular disease, vascular risk profiling and cardiovascular comorbidity, acute treatment and etiologic evaluation, and long-term functional or cognitive outcomes.

As shown in Table 3, the most frequent keywords included stroke (1,999), ischemic stroke (1,333), risk factors (1,101), risk (630), and disease (534). High-betweenness centrality terms included complications (0.08), cerebrovascular disease (0.06), events (0.06), atherosclerosis (0.06), and prevalence (0.05). These bridging terms connected epidemiologic, cardiovascular, diagnostic, and outcome-oriented lines of research.

**Table 3:** Top 10 high-frequency keywords and top 10 keywords with high betweenness centrality.

Rank	High-frequency keyword	Frequency	High-centrality keyword	Betweenness centrality
1	Stroke	1,999	Complications	0.08
2	Ischemic stroke	1,333	Cerebrovascular disease	0.06
3	Risk factors	1,101	Events	0.06
4	Risk	630	Atherosclerosis	0.06
5	Disease	534	Prevalence	0.05
6	Mortality	502	Myocardial infarction	0.05
7	Young adults	428	Follow up	0.05
8	Prevalence	402	Coronary heart disease	0.05
9	Clinical outcomes	388	Magnetic resonance imaging	0.05
10	Adults	384	Diagnosis	0.04

Keyword variants were standardized before ranking. Betweenness centrality values were generated using CiteSpace.

Keyword clustering further clarified the thematic structure (Figure 5B). In the CiteSpace clustering map, nodes were displayed with uniform size, and links indicated co-occurrence relationships. Cluster quality was assessed using modularity (*Q*) and silhouette (*S*) values. The modularity *Q* value was 0.3734, and the weighted mean silhouette *S* value was 0.6917. Based on conventional interpretation criteria, these values indicated a significant and reasonably coherent clustering structure. The principal clusters were #0 risk factors, #1 rehabilitation, #2 intravenous thrombolysis, #3 patent foramen ovale, #4 Fabry disease, and #5 cerebral venous thrombosis. Cluster #0 represented the vascular and epidemiologic line of the field. Cluster #1 represented functional recovery and post-stroke care. Cluster #2 highlighted acute treatment-related research. Cluster #4 pointed to rare vascular and genetic conditions. Clusters #3 and #5 reflected etiologic evaluation and less common stroke mechanisms in younger patients.

Temporal analyses showed a shift in research emphasis (Figures 5C, D). Early burst keywords mainly reflected classical cerebrovascular, thrombotic, and etiologic topics. Representative examples included cerebral infarction, cerebral ischemia, transesophageal echocardiography, venous thrombosis, and cerebrovascular disease. Subsequent bursts involved cardiovascular, genetic, and registry-based topics, such as myocardial infarction, factor V Leiden, mutation, trial, Fabry disease, and registry. Recent bursts included medical therapy, health care professionals, disease burden, mechanical thrombectomy, and case reports. The timezone visualization showed a similar transition. Earlier studies were dominated by ischemic stroke, risk factors, cerebral infarction, patent foramen ovale, cardiovascular disease, myocardial infarction, and cerebral ischemia. Later years showed the emergence of disease burden, mechanical thrombectomy, medical therapy, care, stroke survivors, quality of life, fatigue, systematic review, machine learning, social support, unmet needs, caregivers, and functional outcomes. Overall, the field gradually expanded from traditional vascular, diagnostic, and etiologic concerns to acute treatment, disease-burden assessment, survivorship, and long-term outcome research.

### PubMed external validation

External validation used an independent PubMed dataset constructed with the same conceptual search logic. The PubMed validation screening pool contained 8,094 records after article-type filtering. After eligibility screening, 2,929 records were retained. Annual publication trends in PubMed were highly consistent with those in the full WoSCC dataset across 1996–2026. Spearman's ρ was 0.926 (*P* = 8.87 × 10^−14^), and Pearson *r* was 0.914 (*P* = 7.36 × 10^−13^). Because PubMed served as an independent external validation dataset, retrieval counts were summarized descriptively rather than treated as search-field sensitivity reductions (Table 4).

**Table 4:** Retrieval-level external validation and search-strategy sensitivity analysis.

Strategy	Included records	Change vs. full WoSCC	Spearman's ρ with full WoSCC	Pearson *r* with full WoSCC
Full WoSCC topic-field strategy	5,249	Reference	Reference	Reference
PubMed external validation	2,929	—	0.926; *P* = 8.87 × 10^−14^	0.914; *P* = 7.36 × 10^−13^
Strict WoSCC title-field subset	1,667	−68.2%	0.960; *P* = 1.61 × 10^−17^	0.963; *P* = 3.88 × 10^−18^

Included records refer to eligible records retained after database filtering and screening. PubMed was used as an independent external validation dataset; therefore, record-count differences between PubMed and WoSCC were not interpreted as sensitivity-related loss. The percentage change was calculated using included records. Spearman's ρ and Pearson r were calculated using paired annual publication counts from 1996 through 2026.

**Figure 6:**
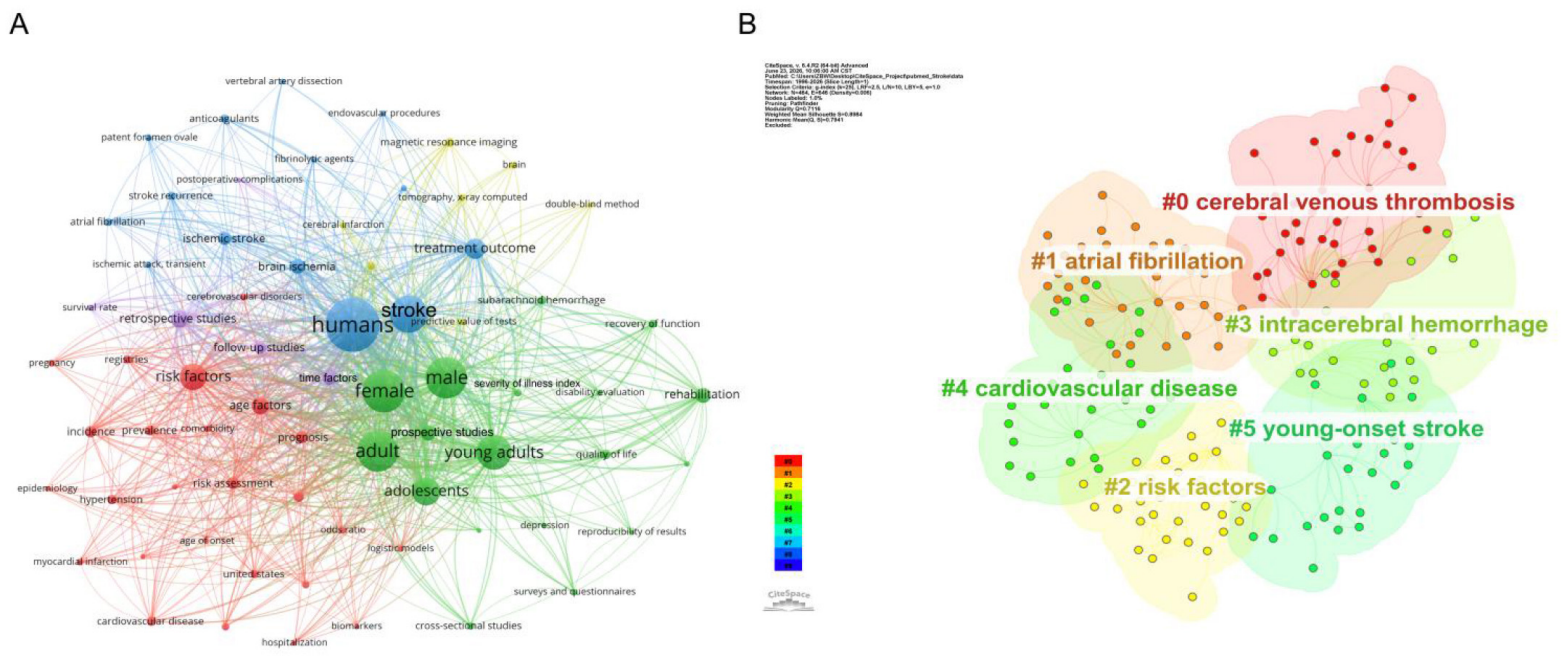
PubMed external validation of the keyword structure. **(A)** VOSviewer keyword co-occurrence network based on standardized PubMed records; node size reflects keyword occurrence, colors indicate co-occurrence clusters, and links indicate keyword co-occurrence relationships. **(B)** CiteSpace keyword clustering map based on PubMed records. Quantitative overlap and similarity metrics are summarized in Table 5.

At the keyword level, the standardized PubMed keyword co-occurrence network is shown in Figure 6A. The CiteSpace keyword clustering map shown in Figure 6B had a modularity Q value of 0.7116 and a weighted mean silhouette S value of 0.8984, indicating a well-structured validation network with clear modular organization and high cluster homogeneity. Six major PubMed keyword clusters were identified in Figure 6B: #0 cerebral venous thrombosis, #1 atrial fibrillation, #2 risk factors, #3 intracerebral hemorrhage, #4 cardiovascular disease, and #5 young-onset stroke. Quantitative comparison based on standardized keywords showed that 14 of the top 30 keywords overlapped between WoSCC and PubMed. The top-30 overlap rate was 46.7%, the Jaccard similarity was 0.304, Spearman's ρ was 0.596 (*P* = 0.025), and the cosine similarity was 0.754. For the top 50 keywords, 20 terms overlapped. The top-50 overlap rate was 40.0%, the Jaccard similarity was 0.250, Spearman's ρ was 0.547 (*P* = 0.012), and the cosine similarity was 0.752 (Table 5). Shared high-frequency terms included ischemic stroke, risk factors, young adults, clinical outcomes, patent foramen ovale, atrial fibrillation, intracerebral hemorrhage, mechanical thrombectomy, and blood pressure. These findings indicate that the main WoSCC-derived temporal and thematic patterns were partly reproduced in PubMed.

**Table 5:** Keyword-level external validation and search-strategy sensitivity analysis.

Comparison	Keyword set	Overlap	Jaccard similarity	Spearman's ρ	Cosine similarity
WoSCC vs. PubMed	Top 30	14/30 (46.7%)	0.304	0.596; *P* = 0.025	0.754
WoSCC vs. PubMed	Top 50	20/50 (40.0%)	0.250	0.547; *P* = 0.012	0.752
Full WoSCC vs. strict title-field subset	Top 30	24/30 (80.0%)	0.667	0.884; *P* = 9.90 × 10^−9^	0.938
Full WoSCC vs. strict title-field subset	Top 50	41/50 (82.0%)	0.695	0.805; *P* = 2.16 × 10^−10^	0.947

Keyword-level comparisons were based on standardized keyword frequencies. PubMed was used for external validation, whereas the strict title-field subset was used for search-strategy sensitivity analysis. Overlap indicates the number and percentage of shared terms within the top-30 or top-50 keyword sets. Spearman's ρ was calculated among shared high-frequency keywords.

### Search-strategy sensitivity analysis

Search-strategy sensitivity was evaluated by comparing the full WoSCC topic-field dataset with a strict title-field subset. The full WoSCC strategy retrieved 8,966 records, of which 5,249 were included. The strict title-field strategy retrieved 3,908 records and retained 1,667 eligible records, representing a 68.2% reduction compared with the full WoSCC dataset. Despite this reduction, annual publication trends remained highly consistent. Spearman's ρ was 0.960 (*P* = 1.61 × 10^−17^), and Pearson *r* was 0.963 (*P* = 3.88 × 10^−18^; Table 4).

Keyword-level sensitivity analysis showed similar results. Using standardized keyword frequencies, the full WoSCC dataset and the strict title-field subset shared 24 of the top 30 keywords. The top-30 overlap rate was 80.0%, the Jaccard similarity was 0.667, Spearman's ρ was 0.884 (*P* = 9.90 × 10^−9^), and the cosine similarity was 0.938. For the top 50 keywords, 41 terms overlapped. The top-50 overlap rate was 82.0%, the Jaccard similarity was 0.695, Spearman's ρ was 0.805 (*P* = 2.16 × 10^−10^), and the cosine similarity was 0.947 (Table 5). Overlapping terms included stroke, ischemic stroke, risk factors, young adults, young-onset stroke, acute ischemic stroke, patent foramen ovale, cardiovascular disease, rehabilitation, cryptogenic stroke, atrial fibrillation, hypertension, cerebrovascular disease, intracerebral hemorrhage, and atherosclerosis. These findings indicate that the main temporal trends and thematic structures were robust to a stricter title-field search strategy.

### Academic influence and intellectual base

Academic influence was reflected in highly cited publications and co-cited references. The most cited retrieved publications covered global stroke burden, stroke statistics, subtype classification, epidemiology in young adults, vascular risk factors, prognosis, recurrence, and long-term consequences. Among retrieved publications, Benjamin et al. (2017) in Circulation ranked first with 7,078 citations (Table 6), followed by Feigin et al. (2014) in The Lancet with 3,036 citations, Katan and Luft (2018) in Seminars in Neurology with 1,686 citations, van Gijn et al. (2007) in The Lancet with 1,449 citations, and Feigin et al. (2024) in The Lancet Neurology with 1,061 citations. Other highly cited publications included studies on metabolic risk, interatrial septal abnormalities, pediatric stroke management, reversible cerebral vasoconstriction syndromes, and the Helsinki Young Stroke Registry.

**Table 6:** Top 10 most-cited publications in the retrieved WoSCC dataset.

Rank	Citations	Journal	Title	First author	Year	DOI
1	7,078	Circulation	Heart Disease and Stroke Statistics-2017 Update A Report From the American Heart Association	Emelia J. Benjamin	2017	10.1161/CIR.0000000000000485
2	3,036	Lancet	Global and regional burden of stroke during 1990–2010: findings from the Global Burden of Disease Study 2010	Valery L. Feigin	2014	10.1016/S0140-6736(13)61953-4
3	1,686	Semin Neurol	Global Burden of Stroke	Mira Katan	2018	10.1055/s-0038-1649503
4	1,449	Lancet	Subarachnoid hemorrhage	Jan van Gijn	2007	10.1016/S0140-6736(07)60153-6
5	1,061	Lancet Neurol	Global, regional, and national burden of stroke and its risk factors, 1990–2021: a systematic analysis for the Global Burden of Disease Study 2021	Valery L. Feigin	2024	10.1016/S1474-4422(24)00369-7
6	880	Lancet	Metabolic mediators of the effects of body-mass index, overweight, and obesity on coronary heart disease and stroke: a pooled analysis of 97 prospective cohorts with 1.8 million participants	Yuan Lu	2014	10.1016/S0140-6736(13)61836-X
7	725	Neurology	Interatrial septal abnormalities and stroke - A meta-analysis of case-control studies	James R. Overell	2000	10.1212/WNL.55.8.1172
8	723	Stroke	Management of Stroke in Infants and Children a Scientific Statement from a Special Writing Group of the American Heart Association Stroke Council and the Council on Cardiovascular Disease in the Young	E. Steve Roach	2008	10.1161/STROKEAHA.108.189696
9	625	Ann Intern Med	Narrative review: Reversible cerebral vasoconstriction syndromes	Leonard H. Calabrese	2007	10.7326/0003-4819-146-1-200701020-00007
10	621	Stroke	Analysis of 1008 Consecutive Patients Aged 15 to 49 with First-Ever Ischemic Stroke the Helsinki Young Stroke Registry	Jukka Putaala	2009	10.1161/STROKEAHA.108.529883

Citation counts refer to WoSCC citation counts for publications included in the retrieved dataset.

The co-cited reference analysis revealed the field's intellectual knowledge base. The modularity *Q* value was 0.8604, and the weighted mean silhouette *S* value was 0.9265. Table 7 shows that Adams et al. (1993) was the most frequently co-cited reference, with 548 co-citations. It was followed by Putaala et al. (2009; 293), Kissela et al. (2012; 191), Schievink (2001; 165), and Ekker et al. (2018; 143). These co-cited references anchored the field around stroke subtype classification, young-onset stroke registries, temporal epidemiology, cervical-artery dissection, prognosis, and long-term consequences.

**Table 7:** Top 10 most frequently co-cited references in the reference co-citation analysis.

Rank	Reference	Co-citations	Journal	First author	Year	DOI
1	Classification of subtype of acute ischemic stroke. Definitions for use in a multicenter clinical trial. TOAST. Trial of Org 10172 in Acute Stroke Treatment	548	Stroke	Harold P. Adams	1993	10.1161/01.STR.24.1.35
2	Analysis of 1008 consecutive patients aged 15 to 49 with first-ever ischemic stroke: the Helsinki young stroke registry	293	Stroke	Jukka Putaala	2009	10.1161/STROKEAHA.108.529883
3	Age at stroke: temporal trends in stroke incidence in a large, biracial population	191	Neurology	Brett M. Kissela	2012	10.1212/WNL.0B013E318270401D
4	Spontaneous Dissection of the Carotid and Vertebral Arteries	165	N Engl J Med	Wouter I. Schievink	2001	10.1056/NEJM200103223441206
5	Epidemiology, etiology, and management of ischaemic stroke in young adults	143	Lancet Neurol	Merel S. Ekker	2018	10.1016/S1474-4422(18)30233-3
6	Ischaemic stroke in young adults: risk factors and long-term consequences	125	Nat Rev Neurol	Noortje A.M.M. Maaijwee	2014	10.1038/NRNEUROL.2014.72
7	Cervical-artery dissections: predisposing factors, diagnosis, and outcome	124	Lancet Neurol	Stéphanie Debette	2009	10.1016/S1474-4422(09)70084-5
8	Clinical outcome in 287 consecutive young adults (15 to 45 years) with ischemic stroke	123	Neurology	Didier Leys	2002	10.1212/WNL.59.1.26
9	Ischaemic stroke in young adults: predictors of outcome and recurrence	119	J Neurol Neurosurg Psychiatry	Krassen Nedeltchev	2005	10.1136/JNNP.2004.040543
10	Epidemiology and etiology of ischemic stroke in young adults aged 18 to 44 years in northern Sweden	118	Stroke	Bjarne Winther Kristensen	1997	10.1161/01.STR.28.9.1702

Co-citation counts refer to the frequency with which each reference was co-cited with other references within the retrieved WoSCC dataset and should be distinguished from WoSCC citation counts for retrieved publications.

**Figure 7:**
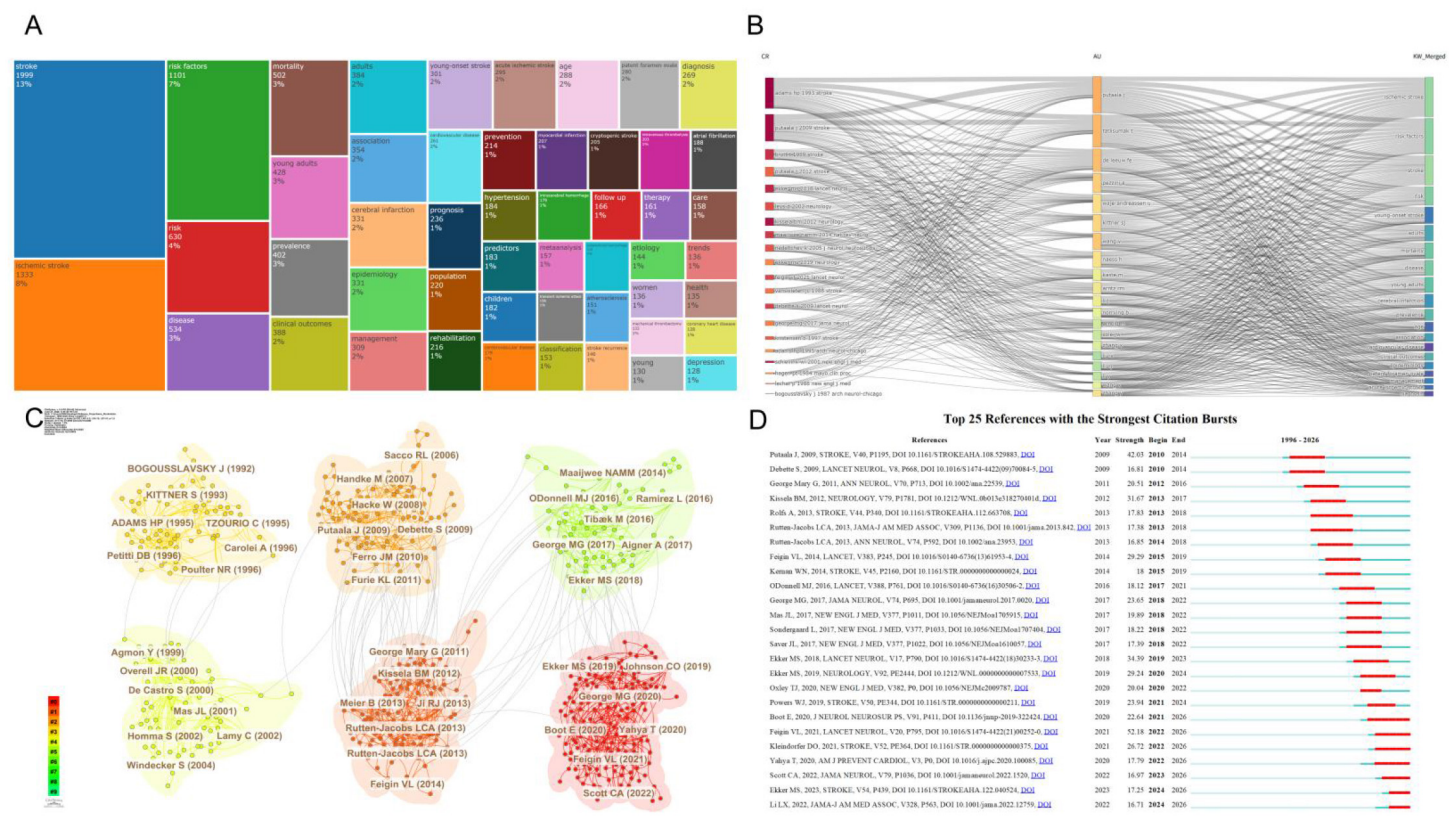
Citation-based knowledge structure and intellectual base in young-onset stroke research. **(A)** Treemap overview of thematic distribution; rectangle size reflects keyword frequency. **(B)** Alluvial map linking co-cited references, core authors, and merged keywords; band width reflects the relative frequency of displayed associations. **(C)** CiteSpace reference co-citation clustering network; links indicate co-citation relationships among references. **(D)** Top 25 references with the strongest citation bursts; red bars indicate burst periods.

The co-citation network in Figure 7C showed several temporally differentiated knowledge communities. Gray lines indicate co-citation links among references and show how temporally separated knowledge communities were connected. Earlier clusters were organized around classic clinical and epidemiologic studies from the 1990s and early 2000s. A subsequent community centered on registry, etiologic, and arterial-dissection studies. A mid-period community reflected increasing attention to temporal trends, risk factors, prognosis, and long-term consequences. Recent nodes indicated expansion toward global burden, recurrent stroke risk, post-stroke cognitive outcomes, and survivorship-oriented research.

Reference burst analysis further showed changing academic focus over time (Figure 7D). The strongest burst was observed for Feigin et al. (2021), with a burst strength of 52.18 during 2022–2026. Other strong bursts included Putaala et al. (2009; 42.03; 2010–2014), Ekker et al. (2018; 34.39; 2019–2023), Kissela et al. (2012; 31.67; 2013–2017), Feigin et al. (2014; 29.29; 2015–2019), and Ekker et al. (2019; 29.24; 2020–2024). Recent burst references that remained active through 2024–2026 included studies related to guideline-based management, global burden, recurrent risk, cognitive outcomes, and survivorship. As supplementary visualizations, Figure 7B links cited references, authors, and merged keywords, whereas Figure 7A provides a treemap-style overview of thematic distribution.

## Discussion

### Principal findings and interpretation

This bibliometric analysis provides an overview of global research on young-onset stroke from 1996 through 2026. The study included 5,249 WoSCC publications identified through a topic-field search and prespecified eligibility screening. Publication output showed a phased upward pattern: it increased modestly during 1996–2006, rose further during 2007–2018, and entered a higher-output phase from 2019 to 2025. The highest annual output was observed in 2022, and productivity remained high through 2025. The 2026 count should be interpreted as a partial-year value. This growth is consistent with increasing clinical and public health recognition that stroke in younger adults is not rare and may carry a long-term burden across decades of life (5, 13).

The analytical design separated the primary WoSCC bibliometric dataset from PubMed external validation and strict title-field sensitivity analysis. PubMed validation showed highly consistent annual publication trends compared with WoSCC, and keyword-level comparisons partly reproduced the main WoSCC-derived thematic structure. The strict title-field sensitivity analysis showed strong agreement with the full WoSCC topic-field dataset, indicating that the main temporal and thematic patterns were not primarily driven by the broader topic-field retrieval. These analyses support the robustness of the main bibliometric patterns while maintaining a clear distinction between external database validation and search-strategy sensitivity analysis.

The knowledge structure identified in this study was organized around ischemic stroke and cerebrovascular disease. Other central themes included vascular risk profiling, cardiovascular comorbidity, acute treatment, etiologic evaluation, and long-term functional or cognitive outcomes. These themes are broadly consistent with contemporary reviews and cohort-based studies of young stroke, which emphasize heterogeneous etiologies, conventional vascular risk factors, and long-term prognosis in this population (2, 4). The bibliometric findings should be interpreted as patterns of research activity and intellectual organization, not as direct clinical evidence of causality. For example, the prominence of patent foramen ovale, arterial dissection, cerebral venous thrombosis, and Fabry disease in the keyword and co-citation networks indicates sustained scholarly attention to etiologic evaluation in younger patients. It does not quantify patient-level causal effects or etiologic proportions.

### Field growth, global distribution, and collaboration

The expansion of young-onset stroke research should be viewed within the broader context of changing stroke epidemiology (14). Age-specific analyses further show that stroke incidence has declined or stabilized in some older populations, whereas in younger age groups it has increased or declined less rapidly (15, 16). Global burden analyses have also highlighted that adolescents and young adults account for a substantial share of stroke burden, particularly in low- and middle-income settings (7, 13). Against this background, the growth in publication output suggests that young-onset stroke has moved from a relatively specialized topic to a more visible area within vascular neurology and public health.

The geographic and institutional distribution of research remained uneven. The United States and China were the largest contributors, and major European, North American, and Asia-Pacific research groups also occupied prominent positions in collaboration networks. Although participation has broadened internationally, publication output remains concentrated in countries and institutions with stronger research infrastructure. This imbalance is important because young stroke burden is not confined to high-income countries. Previous studies have shown that younger stroke age and limited access to organized stroke care remain major challenges in low- and middle-income countries (17, 18). China-specific stroke statistics further show why national surveillance capacity is relevant when interpreting Chinese research output (19). Future collaboration should improve the representation of under-studied regions, support harmonized data collection, and strengthen multicenter young-onset stroke registries across diverse healthcare systems.

The institutional and author networks showed that the field is shaped by several established research groups rather than by a fully integrated global network. Core contributors from Nordic, Dutch, North American, Chinese, Italian, and other European groups have provided major registry-based, epidemiological, and long-term outcome studies (4, 20). This concentration has helped build a coherent intellectual base. It also highlights the need for broader cross-regional collaboration, especially in regions where young stroke burden is high but bibliometric visibility remains limited.

### Thematic evolution and intellectual base

The keyword and co-citation analyses suggest that young-onset stroke research has evolved through several overlapping phases. Earlier work focused on classical cerebrovascular disease, cerebral infarction, thrombotic mechanisms, stroke classification, and etiologic work-up. This pattern is consistent with foundational studies on stroke subtype classification, early young-stroke cohorts, and epidemiological investigations (4, 21). Subsequent work increasingly emphasized registry-based research, temporal trends, vascular risk factors, recurrence, and long-term prognosis (15, 22).

In the keyword clustering, the principal themes were risk factors, rehabilitation, intravenous thrombolysis, patent foramen ovale, Fabry disease, and cerebral venous thrombosis. These clusters delineate research communities centered on vascular risk, rehabilitation, reperfusion therapy, and etiologic evaluation. Clusters labeled “patent foramen ovale” or “Fabry disease” reflect sustained scholarly attention to these topics rather than estimates of their patient-level prevalence or causal contribution.

The co-citation analysis further showed that the field's intellectual base is anchored by several areas, including stroke subtype classification, young-onset stroke registries, temporal epidemiology, cervical-artery dissection, prognosis, and long-term consequences. Highly co-cited references such as the Helsinki Young Stroke Registry, studies of age-at-stroke trends, and reviews of ischemic stroke in young adults have provided conceptual frameworks for the field (4, 6). In parallel, highly cited burden, statistics, and guideline-related papers show that young-onset stroke research is embedded within the broader global stroke and cardiovascular disease literature (1, 23). This dual structure explains why the field is shaped by both specialized young-stroke cohorts and broader stroke-burden and guideline publications.

Recent burst and timezone analyses indicate that the field has expanded toward several emerging themes, including disease burden, medical therapy, mechanical thrombectomy, care, stroke survivors, quality of life, fatigue, social support, unmet needs, caregivers, machine learning, and functional outcomes. These patterns suggest a shift from early etiologic classification and vascular-risk research toward acute treatment, long-term survivorship, and patient-centered outcomes. This transition is consistent with increasing recognition that young stroke survivors often face decades of vascular risk, cognitive and psychological sequelae, social disruption, and employment challenges (24, 25). Together, these trends show that the field has expanded beyond etiologic classification and vascular risk to include acute care, survivorship, and patient-centered outcomes.

### Risk-factor, etiologic, and treatment-related research

Traditional vascular risk factors remained central in the keyword structure. Focused reviews and multinational analyses show that hypertension, smoking, diabetes, dyslipidemia, obesity, and other conventional risk factors are common among young adults with stroke, although their distribution and relative importance vary across regions and etiologic subtypes (26, 27). The persistence of “risk factors,” “cardiovascular disease,” “myocardial infarction,” “coronary heart disease,” and “blood pressure” among high-frequency or high-centrality terms indicates a major organizing line centered on cardiovascular risk profiling in young-onset stroke research. This pattern aligns with young-adult stroke prevention literature emphasizing early risk-factor control and age-specific prevention strategies (28). Region-specific studies from young-adult populations also illustrate how ischemic stroke subtypes and risk-factor profiles vary across health systems (29). In addition, broader prevention literature supports the relevance of conventional, genetic, and occupational vascular risk factors to stroke prevention (30, 31).

The maps also highlight the sustained importance of etiologic evaluation in younger patients. Patent foramen ovale, cervical or cerebral arterial dissection, cerebral venous thrombosis, antiphospholipid antibodies, systemic lupus erythematosus, Factor V Leiden, and Fabry disease appeared in cluster, burst, or co-citation patterns. These topics reflect the broader diagnostic spectrum of young stroke, in which uncommon mechanisms and age-specific conditions may receive more attention than in older stroke populations (21, 32). The bibliometric results show that these etiologic topics are frequently studied and influential within the literature. They do not determine their causal weight or clinical prevalence in individual patients.

Treatment-related themes also became more visible in recent years. Intravenous thrombolysis, mechanical thrombectomy, medical therapy, and guideline-related references appeared in the keyword and reference burst analyses. This pattern likely reflects the broader development of acute stroke care and increasing interest in whether evidence from general adult stroke populations applies equally to younger patients (33, 34). PFO closure trials also contributed to the academic influence of the field, as reflected by highly cited and burst references (35, 36). Clinical recommendations regarding reperfusion therapy, PFO closure, antithrombotic selection, or long-term medical therapy should be based on clinical trials and guidelines rather than on bibliometric prominence alone. Treatment optimization and mechanism-tailored secondary prevention have become increasingly prominent research themes, although clinical recommendations remain dependent on trial and guideline evidence.

### Survivorship and long-term outcomes

A notable finding was the emergence of rehabilitation, quality of life, fatigue, cognitive outcomes, caregivers, social support, unmet needs, and functional outcomes as recent or developing themes. These terms are highly relevant to young-onset stroke. The consequences of stroke may affect education, employment, family roles, mental health, and social participation for decades after the acute event. Previous studies have shown that young survivors may achieve good basic physical recovery but still experience important long-term problems, including cognitive impairment, depression, anxiety, fatigue, and return-to-work difficulties (24, 37).

Cognitive and psychosocial outcomes are best interpreted as post-stroke survivorship issues within this bibliometric landscape. Long-term cognitive impairment after young stroke has been reported in cohort studies and systematic reviews (37, 38). Return to work is also closely related to cognitive performance and psychosocial functioning (25, 39). Depression and anxiety are frequent among young stroke survivors and may substantially affect quality of life (24). In addition, recurrence and long-term mortality remain major concerns, reinforcing the need for sustained secondary prevention and long-term follow-up (22, 40). Long-term prognosis, therefore, remains an important dimension of young-stroke survivorship (41). Clinical outcome studies in young adults also support the need to evaluate functional status beyond early survival (42). These findings align with the bibliometric shift from traditional risk-factor and etiologic research toward chronic outcome assessment and survivorship-oriented investigation.

The field's increasing attention to survivors, caregivers, social support, unmet needs, and functional outcomes suggests that future young-stroke research should move beyond acute hospitalization and early disability scales. It should include long-term neuropsychological outcomes, vocational reintegration, family and caregiver burden, quality of life, and health-system support. The recent prominence of these terms supports a growing emphasis on long-term survivorship and health-system needs.

### Implications for research and interpretation

The findings have several implications for interpreting the young-onset stroke literature. The field is now large enough to support more specialized bibliometric and systematic investigations focused on mechanisms, treatment, rehabilitation, cognition, and global disparities. The combination of WoSCC main analysis, PubMed external validation, and strict title-field sensitivity analysis suggests that the main patterns are reasonably robust, although they were not identical across databases. The moderate keyword overlap between PubMed and WoSCC should be interpreted as partial reproducibility rather than complete equivalence, reflecting differences in indexing systems, controlled vocabulary, database coverage, and document classification.

The results also reinforce the importance of careful terminology and data cleaning in bibliometric studies. Search design, keyword standardization, country harmonization, and separation of VOSviewer and CiteSpace indicators are important for improving transparency and reproducibility. Similar bibliometric studies have shown that search fields, normalization rules, indexing systems, and software-specific metrics can substantially shape the resulting knowledge maps (9, 10). Focused bibliometric work in ischemic cerebrovascular disease also illustrates how risk-factor-related topics can be mapped using science-mapping approaches (43). Bibliometric results should therefore be interpreted as structured evidence about publication patterns and research attention rather than as substitutes for clinical epidemiology, randomized trials, or guideline development.

The concentration of publications around risk factors, PFO, cardiovascular comorbidity, mechanical thrombectomy, rehabilitation, and survivorship identifies areas of substantial research activity but does not establish optimal clinical practice. The main practical contribution of this analysis is to identify under-studied regions and topics for targeted clinical, translational, and health-services research.

### Future directions

Future research on young-onset stroke should prioritize several directions. International studies should adopt clearer and more harmonized definitions of young-onset stroke, including age thresholds, stroke subtypes, and minimum reporting standards. Variation in definitions across studies limits direct comparison and affects bibliometric retrieval. Multinational registries and individual-participant data collaborations could help clarify regional differences in risk-factor profiles, etiologic classification, recurrence, and long-term outcomes (4, 26).

Greater representation of low- and middle-income countries is also needed. The present analysis showed broad global participation, but publication output and collaboration networks remained concentrated in a limited number of high-output countries and institutions. Because the burden of young stroke is substantial in low-resource settings, future research should support infrastructure for stroke registries, acute care pathways, rehabilitation services, and long-term follow-up in under-represented regions (18, 44).

Etiologic and mechanistic research should continue with greater methodological specificity. The prominence of PFO, arterial dissection, cerebral venous thrombosis, Fabry disease, antiphospholipid antibodies, and cardiovascular comorbidity in the bibliometric maps suggests sustained interest in etiologic evaluation. Future studies should distinguish research attention from causal inference and use prospective cohorts, standardized diagnostic protocols, genetic and imaging biomarkers, and long-term outcome data to clarify which mechanisms are most relevant to different young-stroke subgroups (21, 32).

Treatment and secondary-prevention research should focus on questions that are particularly relevant to younger patients (45). Recent bursts involving mechanical thrombectomy, medical therapy, and guideline-related references indicate growing attention to treatment-related themes. Younger adults are often under-represented in trials or analyzed only as subgroups. Future work should evaluate acute treatment access, reperfusion outcomes, PFO-related management, and antithrombotic strategies in age-specific cohorts (33, 34). Risk-factor control and recurrence prevention should also be emphasized in young-stroke prevention pathways (46).

Survivorship research should be expanded. The maps show increasing attention to rehabilitation, cognitive outcomes, fatigue, quality of life, social support, unmet needs, caregivers, and functional outcomes. Longitudinal studies should examine cognitive trajectories, mental health, return to work, family roles, caregiver burden, and socioeconomic consequences after young-onset stroke (24, 37). Interventions should be evaluated in pragmatic and health-system-based studies. Such interventions may integrate cognitive rehabilitation, vocational support, psychological care, lifestyle management, and secondary prevention.

Emerging analytical approaches may help advance the field if applied transparently. Machine learning, artificial intelligence, real-world data, and multimodal phenotyping may support risk stratification, outcome prediction, and personalized follow-up strategies (47, 48). These approaches require external validation, explainability, representative datasets, and careful attention to bias. Future bibliometric work should also improve reproducibility by sharing search strategies, screening decisions, thesaurus files, and code where possible.

### Limitations

Several limitations should be acknowledged. WoSCC was used as the primary database, and PubMed was used for external validation rather than for a fully integrated multi-database bibliometric analysis. This design improved transparency and avoided direct merging of databases with different metadata structures. However, publications indexed only in PubMed, Scopus, Google Scholar, Embase, regional databases, or non-English sources may still have been missed. Differences in database coverage, citation indexing, publication-type classification, and keyword systems may influence bibliometric results (11, 12). PubMed validation, therefore, supports the reproducibility of the main thematic structure, but it does not replace a full multi-database citation and collaboration analysis.

The search strategy used a WoSCC topic-field approach and required young- or age-related terms to be logically linked to stroke- or cerebrovascular-related terms. Even so, no search strategy can capture all relevant young-onset stroke publications without some trade-off between sensitivity and specificity. Definitions of young-onset stroke vary across studies, use different age thresholds, and may include pediatric, adolescent, or young-adult populations. Some relevant studies may not have been captured if the young-adult focus or stroke focus was not clearly represented in searchable metadata. Ambiguous records were assessed using titles, abstracts, keywords, publication types, and available metadata according to predefined rules, but residual metadata-level misclassification cannot be excluded.

Bibliometric indicators should be interpreted cautiously. Citation counts, local citations, co-citations, total link strength, betweenness centrality, and burst strength quantify different aspects of scholarly attention and network structure. They do not directly measure study quality, clinical importance, causal evidence, or guideline strength. Older publications may have more time to accumulate citations, whereas recent high-quality studies may be underrepresented in citation-based rankings. Similarly, burst detection reflects rapid increases in attention rather than definitive evidence of clinical relevance.

The results are sensitive to data cleaning and normalization decisions. Author names, institutional affiliations, country/region labels, and keywords were standardized to improve reproducibility. Records indexed as Mainland China, Hong Kong, Macau, or Taiwan were harmonized into the China category for aggregate reporting. However, residual ambiguity in author disambiguation, institutional naming, keyword variants, abbreviations, and software-generated labels may remain. VOSviewer and CiteSpace also use different algorithms and metrics, and their outputs should not be directly equated without a clear definition (9, 10).

PubMed validation and strict title-field sensitivity analysis strengthened the robustness of the study but did not eliminate all uncertainty. PubMed and WoSCC showed highly consistent annual publication trends, but keyword overlap was only partial because indexing systems and biomedical controlled vocabulary differed. The strict title-field subset reproduced the main trends and keyword structure while inevitably reducing retrieval breadth. These analyses therefore support the robustness of the main patterns but should not be interpreted as proof that the dataset is exhaustive.

The study included articles and reviews. It did not analyze gray literature, conference abstracts, dissertations, policy documents, or non-indexed regional publications. This may have reduced coverage of emerging topics, local health-system research, and early-stage findings. In addition, the 2026 publication count was incomplete because the search was conducted before the end of the year. Citation-based indicators for recent publications should therefore be interpreted with caution.

Finally, this was a bibliometric study rather than a clinical epidemiological review or meta-analysis. The findings identify research trends, influential publications, collaboration structures, and thematic evolution. They cannot determine patient-level risk factors, etiologic proportions, treatment efficacy, or clinical recommendations. Topics such as PFO, arterial dissection, cerebral venous thrombosis, mechanical thrombectomy, rehabilitation, and cognitive outcomes should therefore be understood as areas of research attention and knowledge development. Clinical conclusions require dedicated observational studies, randomized trials, systematic reviews, and guideline-based evidence synthesis.

## Conclusion

This bibliometric analysis maps the development of global research on young-onset stroke from 1996 to 2026. The field has expanded substantially, with sustained high productivity in recent years. Research attention was primarily organized around ischemic stroke, risk factors, cardiovascular comorbidity, etiologic evaluation, acute treatment, rehabilitation, and long-term survivorship outcomes. Publication activity was concentrated in several high-output countries, institutions, journals, and author groups. This pattern indicates the need for broader cross-regional collaboration and greater representation of under-studied settings. PubMed external validation and strict title-field sensitivity analysis supported the robustness of the main temporal and thematic patterns. However, keyword-level findings should be interpreted with awareness that database indexing systems and search-field choices can affect keyword frequencies, cluster labels, and overlap metrics. Overall, the findings highlight young-onset stroke as a growing and increasingly multidimensional research area. Future work should prioritize harmonized definitions, multicenter and multinational data resources, age-specific etiologic and treatment studies, long-term cognitive and psychosocial outcomes, and rehabilitation.
