# Supplementary material for: Global research trends and emerging frontiers in young-onset stroke: a bibliometric analysis (1996–2026)
Source: Front Neurol. 2026 Aug 3;17:1849454. doi: 10.3389/fneur.2026.1849454 (PMC13477900; doi:10.3389/fneur.2026.1849454)
Supplement: Supplementary file 1 [file Supplementary_file_1.pdf]

Supplementary Methods: Complete Database Search Strategies

Search date: 12 June 2026 | Coverage: 1996-2026 | Language: English

This file provides the complete database-specific search strategies used for the primary Web of Science Core Collection (WoSCC) analysis, the independent PubMed validation, and the strict WoSCC title-field sensitivity analysis. The three strategies use the same conceptual framework (stroke/cerebrovascular terms combined with young-age terms), while retaining database-specific field tags and proximity syntax.

Overview of the three searches

Database / field	Analytical role	Retrieval and screening counts	Final eligible
PubMed (MeSH + Title/Abstract)	External biomedical validation	27,868 retrieved; 8,094 after publication-type filtering	2,929
WoSCC Topic field (TS)	Primary science-mapping dataset	8,966 retrieved; 6,063 after year/language/document-type filters	5,249
WoSCC Title field (TI)	Strict search-field sensitivity analysis	3,908 retrieved; 1,752 after year/language/document-type filters	1,667

Field tags, operators, and filters

- PubMed: [mh] indicates a Medical Subject Heading (MeSH); [tiab] searches titles and abstracts; [tiab:~n] specifies PubMed proximity searching.
- WoSCC: TS searches the Topic field; TI searches the Title field; NEAR/10 requires the two concept groups to occur within 10 words of each other, in either order.
- The asterisk (*) is used as a truncation symbol where supported by the database.
- WoSCC interface filters were applied after retrieval: publication years 1996-2026, English language, and document types Article or Review.
- PubMed publication-type filtering and subsequent eligibility screening were performed as described in the manuscript. The PubMed date and language limits are included directly in the query.
- The strict TI strategy uses the same terms and Boolean/proximity structure as the main TS strategy; only the WoSCC search field was changed from Topic to Title.

1. PubMed search strategy (external validation dataset)

Database: PubMed.

Search date: 12 June 2026.

Purpose: Independent biomedical validation of annual publication trends and keyword-level thematic patterns.

Limits included in the query: Publication date from 1 January 1996 through 12 June 2026; English language.

Post-search processing: Publication-type filtering, deduplication, and topic eligibility screening were applied separately; PubMed records were not merged with WoSCC records. The core PubMed concept blocks were combined using Boolean AND, while an additional branch used PubMed-specific proximity expressions for selected young, younger, early-onset, and premature formulations. The strategy intentionally prioritized sensitivity, and topical specificity was subsequently ensured through manual eligibility screening.

Exact query

```
(
(
(
"Stroke"[mh]
```

```
OR "Brain Ischemia"[mh]
OR "Brain Infarction"[mh]
OR "Intracranial Hemorrhages"[mh]
OR "Subarachnoid Hemorrhage"[mh]
OR "Sinus Thrombosis, Intracranial"[mh]
OR stroke[tiab]
OR strokes[tiab]
OR "cerebrovascular accident*"[tiab]
OR "cerebrovascular event*"[tiab]
OR "cerebral infarct*"[tiab]
OR "cerebral infarction"[tiab]
OR "brain infarct*"[tiab]
OR "brain infarction"[tiab]
OR "cerebral ischemia"[tiab]
OR "cerebral ischaemia"[tiab]
OR "brain ischemia"[tiab]
OR "brain ischaemia"[tiab]
OR "ischemic stroke"[tiab]
OR "ischaemic stroke"[tiab]
OR "intracerebral hemorrhage"[tiab]
OR "intracerebral haemorrhage"[tiab]
OR "cerebral hemorrhage"[tiab]
OR "cerebral haemorrhage"[tiab]
OR "hemorrhagic stroke"[tiab]
OR "haemorrhagic stroke"[tiab]
OR "subarachnoid hemorrhage"[tiab]
OR "subarachnoid haemorrhage"[tiab]
OR "cerebral venous thrombosis"[tiab]
OR "cerebral venous sinus thrombosis"[tiab]
OR "cerebral sinovenous thrombosis"[tiab]
)
AND
(
"Young Adult"[mh]
OR "young adult*"[tiab]
OR "young people"[tiab]
OR "young patient*"[tiab]
OR "young-onset"[tiab]
OR "young onset"[tiab]
OR "early-onset"[tiab]
OR "early onset"[tiab]
OR "aged 15 49"[tiab:~1]
OR "aged 18 49"[tiab:~1]
OR "aged 18 50"[tiab:~1]
OR "15 49 years"[tiab:~1]
OR "18 49 years"[tiab:~1]
OR "18 50 years"[tiab:~1]
OR "under 50"[tiab:~0]
OR "under age 50"[tiab:~1]
OR "under the age of 50"[tiab:~0]
OR "younger than 50"[tiab:~0]
OR "younger than age 50"[tiab:~0]
OR "younger than the age of 50"[tiab:~0]
)
)
OR
(
"stroke young"[tiab:~10]
OR "stroke younger"[tiab:~10]
OR "stroke early onset"[tiab:~10]
OR "stroke premature"[tiab:~10]
OR "strokes young"[tiab:~10]
OR "strokes younger"[tiab:~10]
OR "strokes early onset"[tiab:~10]
OR "strokes premature"[tiab:~10]
OR "cerebrovascular accident young"[tiab:~10]
OR "cerebrovascular accident younger"[tiab:~10]
OR "cerebrovascular accident early onset"[tiab:~10]
OR "cerebrovascular accident premature"[tiab:~10]
```

```
OR "cerebrovascular event young"[tiab:~10]
OR "cerebrovascular event younger"[tiab:~10]
OR "cerebrovascular event early onset"[tiab:~10]
OR "cerebrovascular event premature"[tiab:~10]
OR "cerebral infarct young"[tiab:~10]
OR "cerebral infarct younger"[tiab:~10]
OR "cerebral infarct early onset"[tiab:~10]
OR "cerebral infarct premature"[tiab:~10]
OR "cerebral infarction young"[tiab:~10]
OR "cerebral infarction younger"[tiab:~10]
OR "cerebral infarction early onset"[tiab:~10]
OR "cerebral infarction premature"[tiab:~10]
OR "brain infarct young"[tiab:~10]
OR "brain infarct younger"[tiab:~10]
OR "brain infarct early onset"[tiab:~10]
OR "brain infarct premature"[tiab:~10]
OR "brain infarction young"[tiab:~10]
OR "brain infarction younger"[tiab:~10]
OR "brain infarction early onset"[tiab:~10]
OR "brain infarction premature"[tiab:~10]
OR "cerebral ischemia young"[tiab:~10]
OR "cerebral ischemia younger"[tiab:~10]
OR "cerebral ischemia early onset"[tiab:~10]
OR "cerebral ischemia premature"[tiab:~10]
OR "cerebral ischaemia young"[tiab:~10]
OR "cerebral ischaemia younger"[tiab:~10]
OR "cerebral ischaemia early onset"[tiab:~10]
OR "cerebral ischaemia premature"[tiab:~10]
OR "brain ischemia young"[tiab:~10]
OR "brain ischemia younger"[tiab:~10]
OR "brain ischemia early onset"[tiab:~10]
OR "brain ischemia premature"[tiab:~10]
OR "brain ischaemia young"[tiab:~10]
OR "brain ischaemia younger"[tiab:~10]
OR "brain ischaemia early onset"[tiab:~10]
OR "brain ischaemia premature"[tiab:~10]
OR "intracerebral hemorrhage young"[tiab:~10]
OR "intracerebral hemorrhage younger"[tiab:~10]
OR "intracerebral hemorrhage early onset"[tiab:~10]
OR "intracerebral hemorrhage premature"[tiab:~10]
OR "intracerebral haemorrhage young"[tiab:~10]
OR "intracerebral haemorrhage younger"[tiab:~10]
OR "intracerebral haemorrhage early onset"[tiab:~10]
OR "intracerebral haemorrhage premature"[tiab:~10]
OR "cerebral hemorrhage young"[tiab:~10]
OR "cerebral hemorrhage younger"[tiab:~10]
OR "cerebral hemorrhage early onset"[tiab:~10]
OR "cerebral hemorrhage premature"[tiab:~10]
OR "cerebral haemorrhage young"[tiab:~10]
OR "cerebral haemorrhage younger"[tiab:~10]
OR "cerebral haemorrhage early onset"[tiab:~10]
OR "cerebral haemorrhage premature"[tiab:~10]
OR "subarachnoid hemorrhage young"[tiab:~10]
OR "subarachnoid hemorrhage younger"[tiab:~10]
OR "subarachnoid hemorrhage early onset"[tiab:~10]
OR "subarachnoid hemorrhage premature"[tiab:~10]
OR "subarachnoid haemorrhage young"[tiab:~10]
OR "subarachnoid haemorrhage younger"[tiab:~10]
OR "subarachnoid haemorrhage early onset"[tiab:~10]
OR "subarachnoid haemorrhage premature"[tiab:~10]
OR "cerebral venous thrombosis young"[tiab:~10]
OR "cerebral venous thrombosis younger"[tiab:~10]
OR "cerebral venous thrombosis early onset"[tiab:~10]
OR "cerebral venous thrombosis premature"[tiab:~10]
OR "cerebral venous sinus thrombosis young"[tiab:~10]
OR "cerebral venous sinus thrombosis younger"[tiab:~10]
OR "cerebral venous sinus thrombosis early onset"[tiab:~10]
OR "cerebral venous sinus thrombosis premature"[tiab:~10]
OR "cerebral sinovenous thrombosis young"[tiab:~10]
```

```
OR "cerebral sinovenous thrombosis younger"[tiab:~10]
OR "cerebral sinovenous thrombosis early onset"[tiab:~10]
OR "cerebral sinovenous thrombosis premature"[tiab:~10]
)
)
AND 1996/01/01:2026/06/12[dp]
AND english[la]
```

2. WoSCC Topic-field search strategy (primary analysis)

Database: Web of Science Core Collection (WoSCC).
Search field: Topic (TS).
Search date: 12 June 2026.
WoSCC edition coverage: All editions available under the Web of Science Core Collection were selected at the time of searching.
Purpose: Primary science-mapping dataset for publication trends, contributors, collaboration networks, sources, keywords, co-cited references, clusters, and bursts.
Interface filters: Publication years 1996-2026; English language; document types Article or Review.
Logical structure: Stroke/cerebrovascular terms were linked to young-age terms by NEAR/10 or by explicit young-stroke phrases; no standalone unlinked young-onset term was used.

Exact query

```
TS=(
  (
    (
      stroke OR strokes OR "cerebrovascular accident*" OR "cerebrovascular event*"
      OR "cerebral infarct*" OR "brain infarct*"
      OR "cerebral ischemia" OR "cerebral ischaemia"
      OR "brain ischemia" OR "brain ischaemia"
      OR "ischemic stroke*" OR "ischaemic stroke*"
      OR "intracerebral hemorrhag*" OR "intracerebral haemorrhag*"
      OR "cerebral hemorrhag*" OR "cerebral haemorrhag*"
      OR "hemorrhagic stroke*" OR "haemorrhagic stroke*"
      OR "subarachnoid hemorrhag*" OR "subarachnoid haemorrhag*"
      OR "cerebral venous thrombosis"
      OR "cerebral venous sinus thrombosis"
      OR "cerebral sinovenous thrombosis"
    )
    NEAR/10
    (
      young OR younger
      OR "young-onset*" OR "young onset*"
      OR "early-onset*" OR "early onset*"
      OR "young adult*" OR "younger adult*"
      OR "young people" OR "young person*"
      OR "young patient*" OR "younger patient*"
      OR "young population*" OR "younger population*"
      OR "aged 15 to 49" OR "aged 18 to 49" OR "aged 18 to 50"
      OR "15-49 year*" OR "18-49 year*" OR "18-50 year*"
      OR "15 to 49 year*" OR "18 to 49 year*" OR "18 to 50 year*"
      OR "under 50" OR "under age 50" OR "under the age of 50"
      OR "younger than 50" OR "younger than age 50" OR "younger than the age of 50"
    )
  )
  OR
  (
    "young stroke*" OR "young adult stroke*"
    OR "young-onset stroke*" OR "young onset stroke*"
    OR "early-onset stroke*" OR "early onset stroke*"
    OR "premature stroke*" OR "pre-mature stroke*"
    OR "stroke in young adult*" OR "stroke among young adult*"
    OR "stroke in younger adult*" OR "stroke among younger adult*"
    OR "stroke in young people" OR "stroke among young people"
    OR "stroke in the young" OR "stroke at a young age"
    OR "young age stroke*"
  )
)
```

3. WoSCC Title-field search strategy (strict sensitivity analysis)

Database: Web of Science Core Collection (WoSCC).
Search field: Title (TI).
Search date: 12 June 2026.
WoSCC edition coverage: All editions available under the Web of Science Core Collection were selected at the time of searching.
Purpose: Strict search-field sensitivity analysis used to test the stability of the main temporal and thematic findings.
Interface filters: Publication years 1996-2026; English language; document types Article or Review.
Relationship to the main search: The term list, Boolean logic, explicit phrases, and NEAR/10 proximity rule were identical to the Topic-field strategy; only TS was replaced by TI.

Exact query

```
TI=(
  (
    (
      stroke OR strokes OR "cerebrovascular accident*" OR "cerebrovascular event*"
      OR "cerebral infarct*" OR "brain infarct*"
      OR "cerebral ischemia" OR "cerebral ischaemia"
      OR "brain ischemia" OR "brain ischaemia"
      OR "ischemic stroke*" OR "ischaemic stroke*"
      OR "intracerebral hemorrhag*" OR "intracerebral haemorrhag*"
      OR "cerebral hemorrhag*" OR "cerebral haemorrhag*"
      OR "hemorrhagic stroke*" OR "haemorrhagic stroke*"
      OR "subarachnoid hemorrhag*" OR "subarachnoid haemorrhag*"
      OR "cerebral venous thrombosis"
      OR "cerebral venous sinus thrombosis"
      OR "cerebral sinovenous thrombosis"
    )
    NEAR/10
    (
      young OR younger
      OR "young-onset*" OR "young onset*"
      OR "early-onset*" OR "early onset*"
      OR "young adult*" OR "younger adult*"
      OR "young people" OR "young person*"
      OR "young patient*" OR "younger patient*"
      OR "young population*" OR "younger population*"
      OR "aged 15 to 49" OR "aged 18 to 49" OR "aged 18 to 50"
      OR "15-49 year*" OR "18-49 year*" OR "18-50 year*"
      OR "15 to 49 year*" OR "18 to 49 year*" OR "18 to 50 year*"
      OR "under 50" OR "under age 50" OR "under the age of 50"
      OR "younger than 50" OR "younger than age 50" OR "younger than the age of 50"
    )
  )
  OR
  (
    "young stroke*" OR "young adult stroke*"
    OR "young-onset stroke*" OR "young onset stroke*"
    OR "early-onset stroke*" OR "early onset stroke*"
    OR "premature stroke*" OR "pre-mature stroke*"
    OR "stroke in young adult*" OR "stroke among young adult*"
    OR "stroke in younger adult*" OR "stroke among younger adult*"
    OR "stroke in young people" OR "stroke among young people"
    OR "stroke in the young" OR "stroke at a young age"
    OR "young age stroke*"
  )
)
```

Methodological note

The WoSCC Topic-field dataset and the PubMed validation dataset were processed separately. The strict WoSCC Title-field dataset was used only for sensitivity analysis. Record counts shown above correspond to the final revised manuscript and are reported to make the search and screening workflow reproducible.
